# Co‐Creation of Interventions to Promote Critical Health Literacy in the Community: Study Protocol

**DOI:** 10.1111/hex.70670

**Published:** 2026-04-10

**Authors:** Sandro Zacher, Martin Kalteis, Gabriele Meyer, Anke Steckelberg, Jana Hinneburg

**Affiliations:** ^1^ Institute of Health, Midwifery and Nursing Science, Medical Faculty of Martin Luther University Halle‐Wittenberg, University Medicine Halle Halle (saale) Germany

**Keywords:** co‐creation, community health, critical health literacy, feasibility study, health disparities, health promotion, participation, stakeholder

## Abstract

**Background:**

A new community centre in a district with a high amount of equity deserving populations in Halle (Saale), Germany provides a space to design and implement health‐related services that reflect the needs and priorities of local residents. Health information often fails to meet existing quality standards. Therefore, strengthening critical health literacy is essential. Co‐creation is a participatory approach that addresses needs by involving community members as equal partners in the joint development of ideas, concepts and interventions. The study aims to co‐create interventions with residents of the community to promote critical health literacy.

**Methods:**

The study will be conducted as an iterative co‐creation process and feasibility study using both qualitative and quantitative methods, based on the PRODUCES+ framework and guided by the UK Medical Research Council (MRC) framework for developing and evaluating complex interventions. In phase I, co‐creators engage in a multi‐step co‐creation process involving participatory workshops and focus group interviews to explore community health needs, information behaviours and to co‐develop tailored interventions. Interest‐holders representing local institutions are continuously involved to ensure contextual relevance and sustainability. The co‐creation process will be evaluated using the PROSECO framework. In phase II, the developed interventions will undergo feasibility testing and pilot implementation within the newly established community centre, using qualitative and quantitative methods such as think‐aloud, observations, interviews and questionnaires.

**Conclusions:**

The study is expected to develop interventions that will strengthen critical health literacy and empower residents to make informed health decisions. It will also provide insights into mechanisms and success factors of co‐creation.

## 1 Background

Studies indicate that health literacy is a strong predictor of health behaviours, health‐related outcomes and general health status [1, 2, 3, 4]. People showing lower health literacy have consistently been found to have poorer self‐reported health and mental health, higher prevalence of chronic conditions with reduced self‐management capacity, greater engagement in adverse health behaviours, higher hospitalisation rates, increased mortality risk and elevated healthcare utilisation and costs [5, 6, 7].

The integrated conceptual model of health literacy [8] delineates the pathways through which health literacy influences health behaviours and outcomes. It foregrounds four core competencies (accessing, understanding, appraising and applying health information) across the domains of healthcare, disease prevention and health promotion. A key implication of this model is that health literacy is not only an individual resource for navigating health information, but also a prerequisite for meaningful participation in decisions about care and for engaging with health services and policies as an active partner [8]. Concurrently, equity‐deserving groups bear a disproportionate risk of both poorer health and lower levels of health literacy [9, 10, 11].

Despite the existence of ethical standards [12] and patient rights, such as those set out in the German Patient Rights Act of 2013 (Sections 630a–630h of the Civil Code (BGB)), many health information sources [13, 14, 15, 16, 17] do not meet existing quality standards [18, 19]. This highlights the need for rigorous, critical appraisal of their credibility [20, 21]. Since its introduction by Nutbeam [22], critical health literacy has been regarded as an advanced level of health literacy that also encompasses the critical appraisal of health information. While definitions vary, recent conceptual work has identified three core dimensions of critical health literacy: (1) critically appraising health information and claims (e.g. credibility, validity, reliability and applicability), (2) reflecting on the social determinants of health and the power relations that shape health and (3) translating reflection into individual and/or collective action [23, 24, 25, 26, 27]. In this protocol, we build on this integrated understanding and explicitly foreground critical appraisal and individual/collective action as central concerns.

Critical health literacy is discussed as a potential factor for achieving health equity and participation. Knowledge and skills related to empowerment are considered mechanisms in this context [28]. In a recent scoping review, the majority of the identified studies described a positive association between critical health literacy and health behaviour [29]. However, results were heterogeneous and further research should address the remaining issues.

Co‐creation offers a promising approach for tailored solutions that meet the needs of end users and may be more effective and sustainable [30, 31]. There are various definitions of co‐creation. In this study, we use the definition by Vargas et al., which describes co‐creation as continuous, active collaboration and shared decision‐making between various interest groups, with the aim of creative problem‐solving throughout all phases. Following collaborative exploration of the problem, the co‐creation process involves co‐design and co‐production. Co‐design is the co‐development of solutions to the identified problems, while co‐production is the joint implementation of these solutions [31]. We consider co‐creation to be particularly suitable for promoting critical health literacy, as it operationalises person‐centred and participatory mechanisms that are closely aligned with key critical health literacy dimensions. Evidence from a systematic review of health literacy interventions for equity deserving adults suggests that interventions are more likely to be successful when they use person‐centred components such as cultural appropriateness, tailoring, skills building, goal setting and active discussions. The systematic review also highlights participatory approaches, such as community‐based participatory research, as promising for developing health literacy skills and group‐specific interventions for equity deserving groups [10].

Our project is situated in a district with a high amount of equity deserving populations in Halle (Saale) (second largest city in Saxony‐Anhalt, Germany). Its population is highly diverse regarding age, economic status and migration background. This signals the need for increased policy attention to avert negative trends in social cohesion, population health and the local care landscape. These conditions underscore the necessity of targeted, tailored interventions to strengthen critical health literacy within this population, ideally developed and adapted in close collaboration with the community.

A new community centre is under construction in this district. Expansive outdoor grounds and flexible shared spaces are designed to encourage dwell time and facilitate social interaction. With health as a central thematic focus, the centre requires the development of context‐appropriate concepts and services that reflect local priorities and can be implemented sustainably.

## 2 Objectives

The overall aim is to strengthen critical health literacy among residents of the community, enabling them to engage more effectively with health‐related issues and make informed decisions. By promoting critical health literacy, the study seeks to reduce health inequalities and enhance participation.

A co‐creation approach will be used to explore the health‐related needs and priorities of community members. Based on these findings, requirements for the design of tailored health interventions will be identified and interventions to promote critical health literacy will be developed or adapted in collaboration with community members. The co‐creation process itself will be evaluated to identify facilitating and hindering factors. In a subsequent pilot phase, the developed interventions will be tested to assess their feasibility, comprehensibility, comprehension and acceptance. The feasibility of implementing the intervention in the community will also be piloted.

## 3 Methods

### 3.1 Design

The study will be conducted as an iterative co‐creation process [31] and feasibility study employing both qualitative and quantitative methods. The study will be guided overarching by the UK Medical Research Council (MRC) framework for the development and evaluation of complex interventions [32]. As a core component of the study, the co‐creation process will be informed by the PRODUCES+ framework [33, 34]. Additional frameworks will be applied to specific components of the study in line with the PRODUCES+ recommendations.

### 3.2 Study Procedure

**Figure 1:**
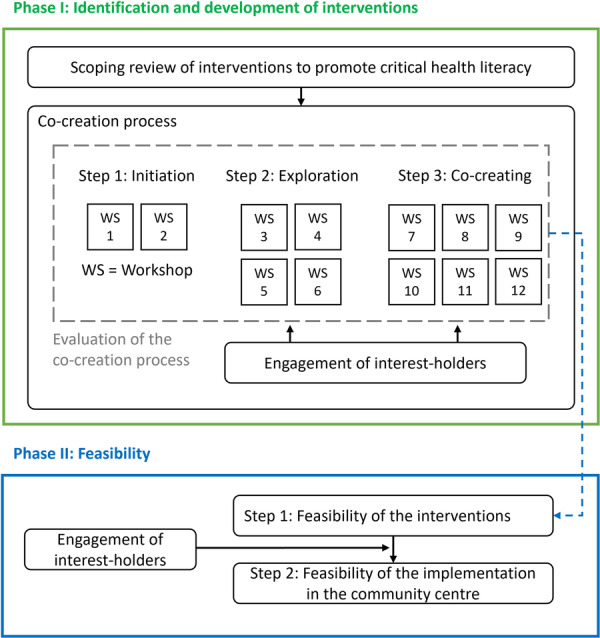
Study procedure.

The study comprises the identification and development of interventions (phase I) as well as the assessment of their feasibility (phase II) [32]. Interest‐holders will be involved in both phases of the study. Due to the colonial connotations of the term ‘stakeholder’ and the potential disrespect it may cause to Indigenous Peoples, we have chosen not to use it [35]. A process evaluation of the co‐creation process will be conducted in parallel, based on the PROSECO framework [36]. The study procedure is summarised in Figure 1.

Due to the flexibility of the co‐creation process and the fact that the interventions will be developed in phase I, the details of phase II will be adapted if necessary. The two phases are described in detail in the following sections.

#### 3.2.1 Phase I: Identification and Development of Interventions

##### 3.2.1.1 Scoping Review

In accordance with the UK MRC framework, a scoping review is being conducted to identify existing interventions that promote critical health literacy. The aim is to inform the development of interventions in Step 3 of the co‐creation process. The review follows the Joanna Briggs Institute guidance [37] and addresses two overarching questions: (1) Which interventions to enhance critical health literacy have been developed and in which target groups and settings have they been implemented? (2) Which barriers, facilitators and research gaps related to promoting critical health literacy are reported?

Eligible records are identified by searching MEDLINE (via PubMed), CINAHL (via EBSCO), APA PsycInfo and PSYNDEXplus (both via Ovid) and ERIC (via IES). In addition, grey literature sources (EThOS, OATD and OpenGrey) are searched. The reference lists of relevant systematic reviews are screened and backward citation tracking of the included studies is undertaken.

The resulting evidence and intervention map provides a robust basis for the co‐creative design of tailored interventions and their adaptation to community settings. The review protocol is registered [38].

##### 3.2.1.2 Co‐Creation Process

The main part of the first phase consists of a multi‐step co‐creation process involving residents of the community, who will hereafter be referred to as co‐creators. For this process, approximately 12 workshops of 120 min each are planned. A key characteristic of successful co‐creation processes is flexibility in implementation [39, 40]. Therefore, the procedure may be adjusted as needed and the number or duration of workshops may be modified in consultation with the co‐creators. The aim of the co‐creation workshops is to enable active participation of the co‐creators. Therefore, we will use methods from an established compendium for co‐creation processes based on the PRODUCES+ framework [34, 41, 42] and evaluate the alignment of these methods with the objectives using the Rainbow framework [43].

Step 1: Initiation of the co‐creation group

In this step, the project objectives, procedures and rules of the co‐creation process are jointly defined. In addition, a shared understanding of the project is developed and group identity is strengthened. Two workshops are planned for this step.

A key element in enabling active participation is to provide co‐creators with the knowledge and skills necessary to contribute effectively to the process. Building on the needs assessment, previously piloted training programmes [44, 45] will be adapted for the target group. The training will be conducted during the second workshop, with the option of extending it to the third workshop if necessary. The training aims to promote critical health literacy among co‐creators, thereby supporting the development of interventions designed to foster it. The focus is on critically appraising information and taking collective action.

Step 2: Exploration of contextual factors and needs assessment

In this step, comprising approximately four workshops, the contextual factors are analysed in accordance with the UK MRC framework and a participatory needs assessment is conducted together with the co‐creators. This will explore (1) the expectations and requirements for a community centre (2) how health‐related issues are currently addressed within the community, (3) which topics are perceived as particularly relevant, (4) how health information is searched for, interpreted and used and (5) how decisions are made based on this information. Prior to the workshops, an exploratory literature search will be conducted to identify existing studies on the needs of the target group in Germany, in order to inform the structure of the workshops.

Based on this exploration, the needs and areas for change required to strengthen critical health literacy will be identified. Furthermore, requirements for future activities within the planned event and communication formats at the community centre will be developed.

Step 3: Co‐creation of the interventions

Based on the findings of the needs assessment, the co‐creators will jointly develop intervention ideas in workshops that directly address the identified needs within the community. These interventions may encompass various approaches to enhance access to and engagement with health information and services. For example, the co‐creators may propose establishing access points to computers that enable residents to search for and critically appraise health information. In addition, they may suggest the provision of tailored informational materials to improve understanding of specific health topics relevant to the community. Group‐based counselling sessions could be developed to support the use of telemedicine, health‐related apps or digital tools for health promotion. Individual counselling and support may also be designed for residents with specific needs, such as guidance on care planning or the use of alert systems in the home environment. In addition, further activities could be developed to promote individual and collective action on specific topics. Examples include dealing with misinformation and disinformation collectively and cooking together in the community centre. Furthermore, citizens could continue to develop the community centre after the project has ended. Furthermore, co‐creators may design and plan workshops and training sessions addressing locally relevant health topics, as well as community events that promote health awareness and participation within the community. A key consideration for all intervention ideas is that they are designed to promote critical health literacy. In the case of health information, this means that the requirements for evidence‐based information are met, facilitating informed decision‐making. In workshops and training courses, attention is paid to balanced and transparent communication. The aspect of critically examining claims or social determinants is also supported. During Step 3, the requirements for the specific design of the interventions to be developed and their implementation in the community centre will be defined.

A range of participatory methods, as proposed by the Health CASCADE network [41, 42] will be employed to iteratively develop and refine these intervention ideas. The results of the scoping review on interventions for promoting critical health literacy will also inform this process. Interventions identified in the scoping review that align with the needs of the co‐creators will be presented, discussed and, if appropriate, adapted following the ADAPT framework [46]. The number of required co‐creation workshops will depend on the number of interventions to be developed or adapted; approximately six workshops are currently planned.

##### 3.2.1.3 Evaluation of the Co‐Creation Process

Process evaluation covers the course of the co‐creation process, its success and how and why certain results were achieved. It enables the systematic observation of process steps, provides insight into mechanisms of action and allows adjustments to be made along the process [36, 47]. The co‐creation process is evaluated using the PROSECO framework [36] during the entire process.

#### 3.2.2 Phase II: Feasibility

Phase II will follow a multi‐step approach, tailored to the specific types of interventions developed during phase I. A logic model for the feasibility phase will be developed based on the identified needs and the interventions to be developed in phase I.

Step 1: Feasibility of the interventions

The feasibility, acceptance, comprehensibility and comprehension of each intervention will be examined with the target group using think‐aloud protocol and focus group interviews.

Step 2: Feasibility of the implementation in the community centre

Once the feasibility of the interventions has been tested, they will be implemented within the community centre for a pilot study to assess their acceptance, uptake, feasibility, comprehensibility, comprehension and potential to improve critical health literacy. The interventions will be evaluated under real‐life conditions using mixed methods. The Consolidated Framework for Implementation Research (CFIR) will serve as the conceptual framework for this implementation phase [48].

#### 3.2.3 Engagement of Interest‐Holders

To explore the contextual factors and prepare for implementation, relevant interest‐holders will be actively involved at different stages throughout the entire study process. This ongoing engagement aims to incorporate interest‐holders' perspectives into the development, adaptation and implementation of the interventions, thereby enhancing the relevance and sustainability of the study outcomes.

### 3.3 Setting

The study will be conducted in a district with a high amount of equity deserving populations in Halle (Saale) (second largest city in Saxony‐Anhalt, Germany). The district comprises four sub‐districts: the northern, southern and western part and the industrial area, the latter of which is scarcely used for residential purposes. The newly established community centre is located in the western part and is easily accessible from all sub‐districts via public transport.

The most recent demographic and socioeconomic data are available from the District Report 2023 [49]. Regarding age distribution, the sub‐districts differ slightly: the average age in the western part (48.1 years) is somewhat higher than the citywide mean, whereas in the southern part (40.9 years) it is lower and in northern part (44.8 years) comparable. The unemployment rate ranges from 13.0% to 16.2%, and the proportion of residents with a migration background varies between 23.7% and 42.3%, both considerably above the city average.

### 3.4 Sample

#### 3.4.1 Phase I: Identification and Development of Interventions

##### 3.4.1.1 Co‐Creators

For the co‐creation process, residents of the community who are interested in health topics and community development will be recruited using purposive sampling based on age, gender, educational background and migration background, aiming to establish a heterogeneous group of co‐creators. A sample of 12 to 15 co‐creators will be targeted to ensure diverse perspectives and to account for potential attrition during the process. Co‐creators who leave the study early will not be replaced in the co‐creation process. We will monitor changes in group composition over time and document potential perspective gaps. These gaps will explicitly inform targeted recruitment in the feasibility phase (phase II).

Inclusion criteria include residence in the district, a minimum age of 18 years and sufficient proficiency in the German language to enable active participation in the workshops. Furthermore, participants must have provided written informed consent prior to taking part in the study. No specific exclusion criteria will be applied.

##### 3.4.1.2 Interest‐Holders

The sample of interest‐holders will include representatives of institutions, associations or organisations that are actively involved in community development or represent the interests of residents. Purposive sampling will also be applied based on the target groups represented (e.g. families, people with a migration background), with a planned sample size of six to eight interest‐holders. The study team will identify interest‐holders through internet searches, which will be complemented by mapping conducted with co‐creators during the exploration and needs assessment stages.

Eligible participants include individuals who are active in the community through their work or community involvement, are at least 18‐years old, possess sufficient German language skills and have provided written informed consent. No additional exclusion criteria will be applied.

#### 3.4.2 Phase II: Feasibility

##### 3.4.2.1 Residents of the District

To evaluate the feasibility of the interventions, residents of the district will be recruited using purposive sampling based on age, gender, educational background and migration background. The aim is to establish a diverse group that reflects the community's composition. Specific participant characteristics may vary depending on the type and target group of the intervention developed in phase I (e.g. older adults, parents or other subgroups). Inclusion criteria will include residence in the district, a minimum age of 18 years and sufficient German language proficiency to enable active participation. People directly involved in the co‐creation of the intervention will be excluded from this phase.

To assess the feasibility of implementation in the community centre, a convenience sample will be used, including all individuals using the centre's activities. The inclusion criterion is a minimum age of 18 years. Co‐creators can also participate in this part of the feasibility phase.

##### 3.4.2.2 Interest‐Holders

In addition, interest‐holders of local organisations will participate in testing the feasibility of implementation in the community centre. The inclusion criteria for these interest‐holders correspond to those applied in phase I.

### 3.5 Recruitment

#### 3.5.1 Co‐Creators and Residents of the District

To raise awareness of the study within the community, several complementary recruitment strategies will be applied. Information about the study will be displayed on notice boards in residential buildings managed by a cooperating housing association. Additional materials, including posters and flyers, will be distributed in the offices of the housing association and shared through the organisation's social media channels. Flyers will also be handed out during community events, such as lantern parades or community games afternoons. Furthermore, information materials will be disseminated via identified interest‐holders, based on the study team's prior internet search. These include local childcare centres, schools, community centres and sports clubs.

Interested residents can contact the study team using the provided details and register on a contact list from which recruitment will proceed. At least two in‐person information sessions will be announced, during which members of the study team will be available in the community to answer questions and provide additional details about the study. In addition, snowball sampling will be applied among participants to reach further potential co‐creators and participants for phase II. The study team will contact interested individuals. The team and the individual then get to know each other. Information is requested about age, gender, educational background and migration background. Further information about the study will be provided, and any questions will be answered. Recruitment efforts will continue until the goal of achieving a heterogeneous group composition is met. Those who are not suitable for the co‐creation process will be asked if they would like to participate in phase II. To acknowledge their time and contribution, co‐creators will receive a compensation of 30 Euros for each 2‐h workshop they attend. In addition, accident insurance for commuting will be provided. Residents participating in phase II will receive 15 Euros in compensation for taking part in a 1‐h data collection process. The compensation will be provided either by bank transfer or in the form of a multifunctional voucher, depending on the participants’ preferences.

#### 3.5.2 Interest‐Holders

Recruitment of interest‐holders will be conducted through direct contact by telephone or email, during which the study and its objectives will be introduced and participation will be invited. Interest‐holders will also be encouraged to distribute study information materials within their own professional networks.

### 3.6 Data Collection

Data will be collected at various stages using qualitative, participatory and quantitative methods (Table 1).

**Table 1:** Data collection in phase I and phase II.

Aim	Data collection	Timing of data collection
Phase I: Development and identification of interventions (Guiding frameworks: UK MRC [32], Produces+ [34], Rainbow [43], ADAPT [46])		
Identification of relevant health topics, current strategies, requirements and needs for interventions	Qualitative: Semi‐structured focus group interviews and creative, participatory workshop methods	During the co‐creation workshops in Step 2
Phase I: Evaluation of the co‐creation process (Guiding framework: PROSECO [36])		
Evaluation of co‐creation process dimensions (delivery, participation, experiential, context and impact)	Quantitative: Process documentation Feedback forms Qualitative: Semi‐structured focus group interviews Participatory observation	During all workshops After each workshop In workshop 2, 6 and 12 During all workshops
Phase I: Interest‐holders (Guiding frameworks: UK MRC [32], CFIR [48])		
Identification of existing health‐related initiatives, facilitating factors, barriers and requirements for interventions at the community centre	Qualitative: Semi‐structured focus group interviews	After Step 2 in the co‐creation process
Phase II: Feasibility of the interventions(Guiding framework: UK MRC [32])		
Evaluation of acceptance, usability, comprehensibility and comprehension of the interventions	Qualitative: Think‐aloud‐protocols Semi‐structured focus group interviews	When the first prototypes of the interventions have been developed Following the revision of the interventions
Phase II: Feasibility of the implementation in the community centre (Guiding framework: UK MRC [32], CFIR [48])		
Evaluation of feasibility, usability, comprehensibility, comprehension and acceptance of the interventions in the community centre	Quantitative: Process documentation Questionnaires Qualitative: Semi‐structured interviews Participatory observation	After the implementation of the interventions After the implementation of the interventions

#### 3.6.1 Phase I: Identification and Development of Interventions

Data will be obtained through participatory workshops and focus group interviews, designed to explore perspectives of community members. Data collection will encompass identifying health topics considered relevant within the community, strategies for searching for, appraising and interpreting health information and approaches to health‐related decision‐making. Furthermore, the needs and requirements of participants for interventions aimed at strengthening critical health literacy will be examined. All workshops conducted in steps 2 and 3 will be audio‐recorded. In addition, the sociodemographic characteristics of the co‐creators, including age, sex, highest level of education, migration background and occupation, will be documented.

##### 3.6.1.1 Evaluation of the Co‐Creation Process

The evaluation of the co‐creation process will be guided by the PROSECO framework [36], which encompasses five dimensions: delivery, participation, experiential, context and impact. Within these dimensions, 37 components are defined that can be assessed throughout the co‐creation process. It is recommended to evaluate at least one component per dimension. The active involvement of co‐creators in co‐designing the evaluation activities is considered essential to identify the need for adaptation and to jointly develop strategies for implementing change [33]. Accordingly, specific components will be pre‐defined for each dimension, which may be adapted or expanded during the process in consultation with the co‐creators as the project evolves (Supporting Information S1).

Data collection will be conducted continuously throughout the co‐creation process, using a range of complementary methods. A person not involved in conducting the study will conduct participatory observations and process documentation during each workshop. In addition, semi‐structured focus group interviews with co‐creators will be conducted at several points during the co‐creation process. For this purpose, the interview guide and observation protocol of the Health Cascade Group will be adapted and used [50, 51]. The first focus group interview will be held after the first step (initiation of the co‐creator group) to explore the starting point and initial expectations. A second focus group interview will take place after completion of the second step and a third will be conducted at the end of the third step of the co‐creation process.

Moreover, additional focus group interviews may be flexibly added if situations arise during the co‐creation process in which co‐creators wish to reflect on their participation and collaboration with the researchers [33].

At the end of the workshops, a feedback form will be used. This allows participants to rate components of the co‐creation process on a scale of 1 to 5. The feedback form is provided in printed form and the evaluation is anonymous (Supporting Information S2).

##### 3.6.1.2 Interest‐Holders

Data collection with interest‐holders will focus on exploring the existing healthcare‐related services and initiatives available in the district, as well as identifying barriers and facilitating factors for the development and implementation of community‐based interventions. Further aspects will include opportunities for collaboration and requirements regarding infrastructure and funding for interventions to be implemented in the community centre (Supporting Information S3). At different stages of the study, semi‐structured focus group interviews will be conducted with the interest‐holders. The interviews will be audio‐recorded. The first focus group interview will take place following the needs assessment with the co‐creators to capture the perspectives of interest‐holders on the identified needs and potential intervention approaches. In addition, socio‐demographic data (age, sex, professional background and experience) will be collected.

Additional focus group interviews will be conducted later in the study and thematically aligned with the interventions to be developed. These interviews will be designed and focused on themes guided by the CFIR [48] and will be further detailed in accordance with the developed interventions.

#### 3.6.2 Phase II: Feasibility

##### 3.6.2.1 Feasibility of the Interventions

The developed or adapted interventions will undergo a qualitative feasibility study to examine their acceptance, usability, comprehensibility and comprehension among community members. As part of an iterative process of piloting and revision, approximately two to four think‐aloud protocols with probing questions will be conducted for each application, along with at least one semi‐structured group interview with six to eight people. These interviews will be audio recorded.

##### 3.6.2.2 Feasibility of the Implementation in the Community Centre

The interventions will be piloted in the community centre with the target groups and interest‐holders. The involvement of interest‐holders is particularly important for a comprehensive assessment of the role of contextual factors.

The pilot will test the feasibility, usability, comprehensibility, comprehension and acceptance of the interventions using a combination of qualitative and quantitative methods. Semi‐structured interviews with service users and interest‐holders are planned, as well as the collection of usage statistics through process documentation and participatory observation during individual interventions. Depending on the intervention developed, it is also planned to collect data on critical health literacy dimensions from service users via questionnaires after interventions, using validated measurement instruments. The detailed design of the data collection will be finalised during the development of the interventions, in consultation with the co‐creators. Based on the results, concrete optimisation strategies will be developed to specifically address the identified strengths and weaknesses of the services. Finally, the interventions will be adapted and the insights gained will be implemented directly to ensure an offering that is user‐friendly, technically sound and oriented towards the target group.

### 3.7 Data Analysis

Analyses of the baseline characteristics will be descriptive. All qualitative data, including semi‐structured focus group and individual interviews, think‐aloud protocols and participatory observations, will be transcribed verbatim and analysed using Braun and Clarke's thematic analysis [52]. Coding will be performed using MAXQDA and Excel spreadsheets, employing both an inductive and a deductive approach. Deductive codes will be based on the relevant frameworks that guide each study phase. These include the PROSECO framework [36] for evaluating the co‐creation process and the CFIR framework [48] for analysing implementation‐related data. However, this approach will also allow additional themes to emerge inductively from the data. Data analysis will be carried out by two researchers independently. In case of disagreement, consensus will be reached through discussion with a third person. The interviews will be summarised and discussed with the co‐creators. Interim results of the analyses will be summarised and discussed with the co‐creators to support interpretation.

The quantitative data, derived from process documentation, feedback forms and questionnaires, will be analysed descriptively to summarise key indicators of feasibility and the dimensions of co‐creating [36].

A triangulation of findings from qualitative and quantitative data will be conducted to integrate different perspectives and generate a comprehensive understanding of the processes, perceptions and mechanisms underlying the interventions [53]. The results will be discussed with the co‐creators.

## 4 Ethical Considerations

The study will be conducted in accordance with the Declaration of Helsinki and ethical approval was obtained from the ethics committee of the Medical Faculty at Martin Luther University Halle‐Wittenberg (No. 2025‐211). The ethics committee will be informed of any changes in procedure. No sensitive personal data as defined under Article 9 of the General Data Protection Regulation (GDPR) will be collected.

Informed consent will be obtained for data collected during co‐creation workshops, focus group interviews with interest‐holders and the feasibility study in phase II. All data will be analysed in anonymised form. At the start of each workshop, co‐creators will be informed about the type of data to be collected and verbal consent will be reconfirmed. Participants may withdraw at any time during or between workshops. Co‐creators who miss a session may re‐join at a later point in the process.

Data processing will be in accordance with data protection regulations. Data will be stored on password‐protected computers and secure servers. Only members of the research team will have access to the data. No data will be shared with third parties, and results will only be reported in aggregated, anonymised form. Data will be stored in accordance with data protection regulations for a period of 10 years and will then be deleted.

## 5 Study Status

Ethical approval was granted on 25 November 2025. Recruitment of participants for the co‐creation process has begun and is expected to continue until February 2026. The scoping review was registered and started on 25 September, 2025. The study is scheduled to run until March 2028.

## 6 Discussion

The present study will address the need to strengthen critical health literacy within a local community in order to enable more informed engagement with health‐related issues and to facilitate active participation. A co‐creation approach will be used to explore the health‐related needs and priorities of community members, providing the basis for identifying requirements for tailored interventions and for developing or adapting these interventions in collaboration with the community. It is expected that target group‐specific interventions will be developed that will lead to greater critical health literacy among the population and enable them to make informed decisions. In a subsequent pilot phase, the interventions will be assessed for their feasibility, usability, comprehensibility, comprehension and acceptance and the feasibility of implementing them within the community centre will be examined. The entire study process will be accompanied by the involvement of interest‐holders. Involving interest‐holders can increase the relevance and sustainability of the study. In addition, possible obstacles to implementation of the developed interventions could be identified. The co‐creation process itself will be evaluated to determine facilitating and hindering factors.

One strength of the study is the application of co‐creation, thereby increasing the possibility of real‐world impact. In addition, the systematic planning of the co‐creation process using the PRODUCES+ framework and, at the same time, the openness to adjustments during the process are strengths of the study [30].

The study presents several challenges and potential barriers. The challenge will be to find co‐creators and encourage them to participate regularly in the workshops. It is possible that a multi‐part workshop series may be difficult to fit into the everyday lives of community members due to professional or personal commitments. Recruiting and building relationships with interested parties and co‐creators therefore plays a central role and may require more resources than planned. In addition, a key task of the research team is to identify and address potential barriers to participation and communication among the co‐creators. Differences in language, literacy and health literacy demands may influence shared understanding and the joint definition and processing of objectives. The two workshops planned for group formation and the learning opportunities offer a good starting point, but it will be essential to reflect on these during the process and, if necessary, take corrective action. However, one major advantage is that the researchers involved have experience in communicating with a wide variety of target groups. Finally, uncertainties about long‐term sustainability complicate both the development and the implementation of co‐created health interventions (e.g. dependence on external funding) and these must be discussed with interest‐holders.

## 7 Conclusion

The study is expected to produce interventions that will strengthen critical health literacy and empower residents to make informed health decisions. Integrating the study into the process of establishing a community centre and engaging the community in co‐creating the centre and its services enables the development of tailored interventions. This increases the centre's applicability and feasibility, thereby contributing to its success. Based on the PRODUCES+ framework for co‐creation and the UK MRC framework for complex interventions, this study will provide insights into the organisation, barriers and facilitators of co‐creation in this community, as well as the feasibility of implementing critical health literacy interventions in a community centre.

## Author Contributions

**Sandro Zacher:** conceptualization, methodology, writing – original draft, writing – review and editing, visualization. **Martin Kalteis:** conceptualization, methodology, writing – review and editing. **Gabriele Meyer:** conceptualization, methodology, funding acquisition, writing – review and editing. **Anke Steckelberg:** conceptualization, methodology, funding acquisition, writing – review and editing. **Jana Hinneburg:** conceptualization, methodology, writing – review and editing.

## Ethics Statement

Ethical approval was obtained from the ethics committee of the Medical Faculty at Martin Luther University Halle‐Wittenberg (No. 2025‐211). The ethics committee will be informed of any changes in procedure.

## Conflicts of Interest

The authors declare no conflicts of interest.

## Patient or Public Contribution

The present study protocol outlines a co‐creation process in which community residents will collaboratively define needs and develop interventions to promote critical health literacy. These interventions will subsequently be integrated into the community centre. The co‐creators will be involved in developing the interview questions for interest‐holders and will participate in evaluating the co‐creation process by setting additional evaluation dates and determining the outcomes. The results of interviews with interest‐holders, the evaluation of the co‐creation process and the piloting of interventions in phase II will also be discussed with the co‐creators.

## Use of Generative AI

During the preparation of this manuscript, the authors used ChatGPT (OpenAI) and DeepL (DeepL SE) to improve wording, clarity and translation. After using generative AI, the authors reviewed and edited the content as necessary and take full responsibility for the final version of the manuscript.

## Permission to Reproduce Material From Other Sources

No material from other sources has been reproduced in this manuscript.

## Supporting information

Supporting File 1:

Supporting File 2:

Supporting File 3:

## Data Availability

Data sharing not applicable to this article as no datasets were generated or analysed during the current study.
