# Supplementary material for: Co‐Creation of Interventions to Promote Critical Health Literacy in the Community: Study Protocol
Source: Health Expect. 2026 Apr 10;29(2):e70670. doi: 10.1111/hex.70670 (PMC13066911; doi:10.1111/hex.70670)
Supplement: Supplementary file 1 — Supporting File 1: [file HEX-29-e70670-s003.pdf]

Supplement 1: Selected components of the PROSECO framework for evaluating the co-creation process

Concept	Definition	Data collection
Dimension: delivery		
Adaptation	The process of adjusting and reporting changes according to new conditions, circumstances, co-creators feedback, and/or in relation to the original design or protocol.	Documentation
Fidelity	This component evaluates the extent to which the completed process or method solution implementation reflects the original aims, designs or protocol; while acknowledging and accommodating potential changes or refinements over time, which occurred in response to new conditions, circumstances or the co-creator's feedback.	Comparison with protocol, interviews
Transparency	This component evaluates the openness regarding the inputs, outputs, and procedural steps of a method or process. It evaluates how well it ensures clear visibility of its steps, components and how clearly it connects input to outcomes.	Observations
Useful outputs	This component evaluates whether the method or process generates outputs that are useful for the co-creators or the process itself. It also evaluates the diverse range of output formats used for visualising the generated data, acknowledging that different contexts or projects may require distinct formats.	Observations, interviews
Dimension: participation		
Motivation	This component evaluates the underlying reasons for why co-creators engage in co-creation; uncovering what simulates and energises them to act. For instance, the reasons they are engaging, the incentives that influenced their decision to join the process, or reasons for staying engaged throughout the process.	Interviews
Retention	This component evaluates the degree to which, and ability to, maintain adequate levels of participation throughout the co-creation process.	Observations
Attendance	This component evaluates the presence of co-creators throughout the co-creation process.	List of participants

Recruitment	This component evaluates the strategies employed to attract and enlist co-creators in the process. It evaluates the extent to which recruitment has achieved its intended goals and predetermined objectives. If this is among recruitment's objectives, it may involve assessing the extent to which representativeness and diversity among co-creators was attained.	Documentation
Conflict management	This component evaluates the ability to address, manage, and resolve conflicts among competing interests within the group. It assesses how well the method or process not only handles conflicts but also leverages them to stimulate constructive debate and foster further development. The emphasis is on promoting resolution in a manner that cultivates a harmonious environment, sustains group cohesion, and utilises conflicts as opportunities for growth. It aims to ensure that conflicts are handled constructively, and decisions are reached through consensus, thus promoting a cooperative and positive development process.	Observations
Group dynamics	This component evaluates the ability to foster active engagement, high involvement and the strategic use of personalised and interactive aspects to create a harmonious and productive co-creation environment. The focus is on how the method or process manages the dynamics of the group (e.g. empathy, discussions, trust building) and encourages respectful interactions and cooperation among co-creators.	Observations, interviews
Impactful decision-making	This component evaluates how well the co-creation method or process facilitates a comprehensive and productive decision-making process. It evaluates whether a decision-making process is structured enough to reach significant, Influential or effective results, that is based on agreement among the co-creators.	Observations
Dimension: experiential		
Acceptability	This component evaluates the extent to which the co-creation process and method is perceived as suitable and agreeable by the co-creators and the rest of interest-holders involved. It measures how well the preferences, needs, expectations and standards set by co-creators and interest-holders are met, making it suitable for adoption or implementation.	Interviews
Clarity	This component evaluates the effectiveness in articulating and ensuring a clear understanding of all the collaboration process among the co-creators, including the nature and scope of the task.	Observations

Expectations	This component evaluates the extent to which the co-creators' anticipated outcomes, preferences, needs or expectations were met.	Feedback forms, Interviews
Experience	This component evaluates the co-creators' holistic experience within the method or process. It includes, for example, their comfort level, their perception of success or their enjoyment of the process.	Feedback forms, interviews
Satisfaction	This component evaluates the level of satisfaction and/or dissatisfaction among the co-creators who participated in the process.	Feedback forms, interviews
Dimension: context		
Feasibility	This component evaluates the practical aspects and likelihood of success for implementing a co-creation process within a specific setting, considering available resources, potential challenges, and alignment with interest-holders' needs, perspectives and goals. It encompasses an analysis of factors such as funding, technical infrastructure and time constraints, alongside identification and mitigation of risks and barriers.	Observations, interviews
Dimension: impact		
Personal change	This component evaluates shifts in perspectives, enhancement of skills, strengthening of social connections and other behavioural transformations occurring during the co-creation process; including the employment of specific methods. These changes reflect the individual and collective growth, learning and development resulting from active engagement in the co-creation process. It also includes self-perceived social, cognitive, and behavioural transformations experienced by co-creators.	Observations, interviews
