# Supplementary material for: Co‐Creation of Interventions to Promote Critical Health Literacy in the Community: Study Protocol
Source: Health Expect. 2026 Apr 10;29(2):e70670. doi: 10.1111/hex.70670 (PMC13066911; doi:10.1111/hex.70670)
Supplement: Supplementary file 2 — Supporting File 2: [file HEX-29-e70670-s001.pdf]

Supplement 2: Feedback form

Please tell us how you experienced today's group meeting.

Please rate the extent to which the following statements apply to you.

1. The organisation of the group meeting was as I expected.

☐	☐	☐	☐	☐
Does not apply at all	Hardly applies	Partly applies	Applies well	Applies very well

2. I thought the topics for the group meeting were well chosen.

☐	☐	☐	☐	☐
Does not apply at all	Hardly applies	Partly applies	Applies well	Applies very well

3. I felt comfortable during the group meeting.

☐	☐	☐	☐	☐
Does not apply at all	Hardly applies	Partly applies	Applies well	Applies very well

4. I enjoyed the group meeting.

☐	☐	☐	☐	☐
Does not apply at all	Hardly applies	Partly applies	Applies well	Applies very well

5. I am pleased with the results of the group meeting.

☐	☐	☐	☐	☐
Does not apply at all	Hardly applies	Partly applies	Applies well	Applies very well

6. I am pleased with the cooperation within the group.

☐	☐	☐	☐	☐
Does not apply at all	Hardly applies	Partly applies	Applies well	Applies very well

What can we do better at the next group meeting?

____

____

____

____
