# Supplementary material for: Co‐Creation of Interventions to Promote Critical Health Literacy in the Community: Study Protocol
Source: Health Expect. 2026 Apr 10;29(2):e70670. doi: 10.1111/hex.70670 (PMC13066911; doi:10.1111/hex.70670)
Supplement: Supplementary file 3 — Supporting File 3: [file HEX-29-e70670-s002.pdf]

Supplement 3: Interview guide for interest-holders

- **Attitudes and general situation in the community**
  - How would you describe the attitudes of residents or local institutions towards health issues and participation?
  - What social or structural conditions favour or hinder the successful implementation of the planned services?
- **Promoting factors**
  - What conditions or resources could facilitate the implementation of health services in the community centre?
  - Which existing networks, collaborations or structures in the community could provide support?
- **Barriers to implementation**
  - What factors could hinder the implementation of the planned health services in the community centre?
  - Are there any organisational, financial, structural or communication barriers that could hinder participation or implementation?
- **Comparison with existing services**
  - Are there any similar offers or initiatives with which you already have experience?
  - How do the planned services at the community centre differ from existing health-related activities or services in the community
- **Advantages and challenges of the services offered**
  - What advantages do you see in the planned offerings compared to previous activities?
  - Are there any potential disadvantages, overlaps or challenges that should be taken into account?
- **External influences**
  - Have there been any recent events, political changes or local developments that could affect the implementation of the planned services?
  - How could such developments influence the work of the community centre or cooperation between interest-holders in the community?

- **Need for support and cooperation**
    - What kind of support from other organisations, local authorities or networks would be helpful in order to implement and maintain the services?
    - How could cooperation between your institution and the community centre be strengthened?
- **Networking and cooperation**
    - To what extent does your organisation already collaborate with other actors in the community or exchange information (e.g. with social, cultural or health institutions)?
    - How could these existing relationships or networks be used to integrate the new health services into the existing structures?
- **External expectations or external pressure factors**
    - Are there any external expectations or requirements (e.g. from funding bodies, local authorities or political guidelines) that could influence implementation?
    - How do such factors affect decisions or priorities in your organisation?
- **Infrastructure and underlying conditions**
    - How does the spatial and organisational infrastructure of the community centre (e.g. premises, accessibility and equipment) influence the implementation of the planned services?
    - Do you see any adjustments or improvements that would be necessary to facilitate implementation?
